# The post-2015 development agenda and South–South and triangular cooperation – How the partnership model should be?

**DOI:** 10.1177/1468018115600123b

**Published:** 2015-12

**Authors:** Mami Yamada Sakurai

**Affiliations:** United Nations Office for South-South Cooperation (UNOSSC), USA

**South–South and triangular cooperation as a partnership model for the post-2015 development agenda**

In the Outcome Document of the United Nations Conference on Sustainable Development,^1^ it was decided to create an Open Working Group (OWG) to propose new development goals after 2015 replacing the Millennium Development Goals.^2^ The OWG report specifically mentions South–South and triangular cooperation in the Goal 17 entitled ‘Strengthen the means of implementations and revitalise the global partnership for sustainable development’, under the topic of ‘technology’ and ‘capacity-building’.

The UN Secretary-General issued a synthesis report on the post-2015 sustainable development agenda,^3^ which highlighted the establishment of new institutions of South–South cooperation, such as the BRICS Development Bank and the Asian Infrastructure Investment Bank, as new opportunities to finance investments in sustainable development. It also mentions that

South–South cooperation and the significant efforts of solidarity by emerging economies is encouraging. More countries will need to commit to increasing their contribution to international public financing and set targets and timelines to do so. In turn, South–South technical assistance and the sharing of experiences through regional forums should be promoted.^4^

As such, South–South cooperation features as a way to unite efforts of the Global South for development, through knowledge and technology transfer and mutual capacity-building efforts,^5^ while triangular cooperation involves Southern-driven partnerships between two or more developing countries supported by a developed country(ies)/or multilateral organisation(s) to implement development cooperation programmes and projects.^6^ Here, the role of developed countries and or multilateral organisations could be to provide financial support to South–South cooperation, facilitate match-making among solution seekers and providers, to provide additional options for knowledge and technology based on its own development experience, and share its expertise on management of development cooperation initiatives with triangular cooperation partners.^7^ Triangular cooperation has a clear potential not only to play an important role in sustainable development trajectories, but also to be a model for global partnership for sustainable development for the next 15 years.

In this context, the following three questions arise: what role could regional fora and financing mechanisms potentially play in implementing the SDGs? What experiences of a regional and inter-regional approach can be built on towards these ends? And what does triangular cooperation mean in the context of global partnership for sustainable development?

## Triangular cooperation initiatives in the health sector through regional and inter-regional approaches

Japan has been a champion in promoting and implementing triangular cooperation projects in partner with developing countries since the 1970s. Japan’s policy framework on South–South cooperation, in general, and triangular cooperation in particular, is defined at the highest level by the Official Development Assistance (ODA) charter and at the operational level by JICA’s policy^8^ (Honda, 2014).

As a triangular cooperation supported by Japan in the health sector, a project of Chagas in Central America is a good example of ways in which partnership arrangements between a traditional donor country and developing countries promote regional South–South cooperation to tackle communicable diseases. In 1997, the Central American Initiative for Chagas Disease Control was launched, and Japan International Cooperation Agency started to work in Guatemala in 2000 to support this regional initiative for interrupting Chagas disease transmission. Based on this pilot experience, the project extended to other countries such as El Salvador, Honduras and Nicaragua. The result of intervention in each country was shared through the compilation of a summary of best practices.^9^ Showing promising results,^10^ the key factor of its success is the existence of regional platform and goal accompanied by a donor country alongside regional organisations such as the Pan American Health Organisation (PAHO).

Japan is also scaling up South–South cooperation through regional and inter-regional centres of excellence.^11^ The triangular cooperation between Angola–Brazil–Japan for strengthening health system in Angola is an example of how Angola benefitted from cooperation by the centres of excellence in Brazil (Oswaldo Cruz Foundation and State University of Campinas), leveraged by Japan’s financial and technical support. Brazilian centres of excellence supported improvements in primary and tertiary healthcare in Angola, while Japan introduced its experience on maternal and child health handbooks in partner with the World Health Organization (WHO), United Nations Children’s Fund (UNICEF) and United Nations Population Fund (UNFPA).^12^ In order to promote regional and inter-regional South–South and triangular cooperation, it is important that regional and multilateral organisations identify and work with existing Southern centres of excellence in key development areas so as to make significant contribution to achieving development goals. Traditional donors can join this effort through triangular arrangements.

An initiative promoted by Inter-American Development Bank (2014) is an interesting example of how regional issues are being addressed through South–South and triangular cooperation from the perspective of regional public goods. The Initiative for the Promotion of Regional Public Goods is based on the rationale that the countries of Latin America and the Caribbean share challenges and opportunities for development that can be addressed more effectively and efficiently at a regional level through collective action and cooperation.^13^ The ‘Caribbean Regional Non-Communicable Disease Surveillance System’ under this initiative supported the creation of an interactive, web-based surveillance system for non-communicable diseases (NCDs) in the region, through a regional centre of excellence (University of the West Indies) in collaboration with regional organisations such as PAHO, Caribbean Epidemiology Centre and the Caribbean Community Secretariat.

These centres of excellence have potential to generate regional public goods in partner with regional organisations, whose enhanced role and capacity will be a key element for the global health governance post-2015.

## How to measure the success of partnership?

Each SDG will establish indicators to be measured. How should we measure the South–South and triangular partnership? By the amount of resource mobilised? By the development result achieved? By the win–win–win situation created? More case analysis of triangular cooperation and successful partnership models need to be fed into the discussion about effective SDG implementation. Further analysis on the existing good practices and future potential of regional and inter-regional organisations and partnerships is also needed.

## Notes

1. A/RES/66/288*, reissued on 17 January 2013

2. Para. 248

3. A/69/700

4. Para. 111

5. From the author’s perspective.

6. United Nations (2012) ‘Framework of operational guidelines on United Nations support to South-South and triangular cooperation’, para. 11.

7. Regarding the supporting role of the multilateral system, please see more in Zhou (2013).

8. Regarding the significance of triangular cooperation for the Government of Japan, please see the ODA White Paper 2011, p. 24 (Ministry of Foreign Affairs of Japan (2012)) as well as the Development Cooperation Charter released in February 2015.

9. http://www.jica.go.jp/english/our_work/thematic_issues/health/c8h0vm0000011ls7-att/study_inf_02.pdf

10. Guatemala (in 2008), Nicaragua and Honduras (both in 2011) received IPCA-PAHO international certification for interruption of transmission of Chagas disease by R. Prolixus, and El Salvador received the same certification for elimination of the same vector in 2010.

11. Please see Chandy L, Hosono A, Kharas H, et al., 2013 for Japan’s centres of excellence approach

12. For more information about this project, please see http://www.jica.go.jp/english/publications/j-world/c8h0vm00008wrfb4-att/1409_04.pdf

13. http://www.iadb.org/en/topics/regional-integration/what-is-the-regional-public-goods-program,2803.html
